# Feasibility, pitfalls and results of a structured concept-development phase for a randomized controlled phase III trial on radiotherapy in primary prostate cancer patients

**DOI:** 10.1186/s12885-022-09434-2

**Published:** 2022-03-28

**Authors:** S. K. B. Spohn, S. Adebahr, M. Huber, C. Jenkner, R. Wiehle, B. Nagavci, C. Schmucker, E. G. Carl, R. C. Chen, W. A. Weber, M. Mix, A. Rühle, T. Sprave, N. H. Nicolay, C. Gratzke, M. Benndorf, T. Wiegel, J. Weis, D. Baltas, A. L. Grosu, C. Zamboglou

**Affiliations:** 1 Department of Radiation Oncology, Medical Center, University of Freiburg, Faculty of Medicine. University of Freiburg, Freiburg, Germany; 2 German Cancer Consortium (DKTK). Partner Site Freiburg, Freiburg, Germany; 3 Berta-Ottenstein-Programme, Faculty of Medicine, University of Freiburg, Freiburg, Germany; 4 Clinical Trials Unit, Faculty of Medicine and Medical Center, University of Freiburg, Freiburg, Freiburg, Germany; 5 Division of Medical Physics, Department of Radiation Oncology, Medical Center, University of Freiburg, Faculty of Medicine, University of Freiburg, Freiburg, Germany; 6 Institute for Evidence in Medicine, Medical Center - University of Freiburg, Faculty of Medicine, University of Freiburg, Freiburg, Germany; 7 Bundesverband Prostatakrebs Selbsthilfe e.V, Freiburg, Germany; 8 Department of Radiation Oncology, University of Kansas Cancer Center, Kansas City, KS 5 USA; 9 Department of Nuclear Medicine, Klinikum rechts der Isar, Technische Universität München, Munich, Germany; 10 Department of Nuclear Medicine, Faculty of Medicine, Medical Center, University of Freiburg, Freiburg, Germany; 11 Department of Urology, Faculty of Medicine, Medical Center, University of Freiburg, Freiburg, Germany; 12 Department of Radiology, Faculty of Medicine, Medical Center, University of Freiburg, Freiburg, Germany; 13 Department of Radiation Oncology, University Hospital Ulm, Ulm, Germany; 14 Department of Cancer Self-Help Research, Comprehensive Cancer Center, Medical Center, University Clinic Center, Freiburg, Germany; 15 German Oncology Center, European University of Cyprus, Limassol, Cyprus

## Abstract

**Objective:**

Failure rate in randomized controlled trials (RCTs) is > 50%, includes safety-problems, underpowered statistics, lack of efficacy, lack of funding or insufficient patient recruitment and is even more pronounced in oncology trials. We present results of a structured concept-development phase (CDP) for a phase III RCT on personalized radiotherapy (RT) in primary prostate cancer (PCa) patients implementing prostate specific membrane antigen targeting positron emission tomography (PSMA-PET).

**Materials and methods:**

The 1 yr process of the CDP contained five main working packages: (i) literature search and scoping review, (ii) involvement of individual patients, patients’ representatives and patients’ self-help groups addressing the patients’ willingness to participate in the preparation process and the conduct of RCTs as well as the patient informed consent (PIC), (iii) involvement of national and international experts and expert panels (iv) a phase II pilot study investigating the safety of implementation of PSMA-PET for focal dose escalation RT and (v) in-silico RT planning studies assessing feasibility of envisaged dose regimens and effects of urethral sparing in focal dose escalation.

**Results:**

(i) Systematic literature searches confirmed the high clinical relevance for more evidence on advanced RT approaches, in particular stereotactic body RT, in high-risk PCa patients. (ii) Involvement of patients, patient representatives and randomly selected males relevantly changed the PIC and initiated a patient empowerment project for training of bladder preparation. (iii) Discussion with national and international experts led to adaptions of inclusion and exclusion criteria. (iv) Fifty patients were treated in the pilot trial and in- and exclusion criteria as well as enrollment calculations were adapted accordingly. Parallel conduction of the pilot trial revealed pitfalls on practicability and broadened the horizon for translational projects. (v) In-silico planning studies confirmed feasibility of envisaged dose prescription. Despite large prostate- and boost-volumes of up to 66% of the prostate, adherence to stringent anorectal dose constraints was feasible. Urethral sparing increased the therapeutic ratio.

**Conclusion:**

The dynamic framework of interdisciplinary working programs in CDPs enhances robustness of RCT protocols and may be associated with decreased failure rates. Structured recommendations are warranted to further define the process of such CDPs in radiation oncology trials.

**Supplementary Information:**

The online version contains supplementary material available at 10.1186/s12885-022-09434-2.

## Introduction

Randomized controlled trials (RCTs) are the backbone of evidence-based medicine and are needed to establish novel therapeutic or diagnostic procedures. However, the failure rate for phase II and III trials are approximately 70 and 50%, respectively and can arise from different issues such as safety-problems, underpowered statistics, lack of efficacy, lack of funding or insufficient patient recruitment [1, 2]. This phenomenon is even more pronounced in oncology trials [3]. Considering the impact on the cancer patients’ lives as well as the personal and cost intensiveness of RCTs a reduction of the failure rate is warranted. Failures in RCTs are caused from lacking efficacy, safety issues, lack of funding to complete a trial, as well as problems with patient recruitment, enrollment and retention [2]. All these issues have to be addressed by a robust study protocol, which considers all possible pitfalls. For example, Getz et al. reported that more than 40% of study protocols were amended prior to the first visit, and one third of amendments were avoidable. Protocol amendments lead into unplanned expense, delays and unexpected burden for investigative sites [4]. Consequently, a proper conceptualization and design of a RCT and its protocol is crucial and may avoid delays or even failure of the entire study.

In this manuscript, we present the feasibility, the pitfalls and the results of a concept-development phase (CDP) for a study protocol of a phase III trial on image-guided stereotactic body radiotherapy (SBRT) in primary localized prostate cancer (PCa) patients (HypoFocal-SBRT). The study will compare moderately hypofractionated radiotherapy (MHRT) and focal dose escalated (SBRT) based on information of multimodal diagnostic imaging. The clinical and theoretical rationale of the HypoFocal-SBRT study is based (i) on the current evidence to use SBRT [5, 6] and (ii) the suggested improved tumour control of focal dose escalation [7]. Additionally, (iii) our group and others performed histology-imaging comparison studies, demonstrating that the combined usage of multiparametric magnet resonance tomography (mpMRI) and positron emission tomography targeting the prostate specific membrane antigen (PSMA-PET) improves the PCa detection and provides complimentary spatial information [8–13]. Thus, implementation of both imaging methods into RT planning might significantly affect focal dose escalated regimens, might improve tumor coverage in focal therapy approaches and builds the foundation for the HypoFocal-SBRT trial.

The aim of the one-year lasting CDP process was to finalize a study protocol based on the input of different health care professionals as well as patients and patient representatives. Based on this, five main working programs (WPs) were initiated:

i. Literature search and scoping review
ii. Involvement of individual patients, patients’ representatives and patients’ self-help groups
iii. Involvement of national and international experts and expert panels
iv. A pilot study
v. *In-silico* radiotherapy planning studies

**Fig. 1:**
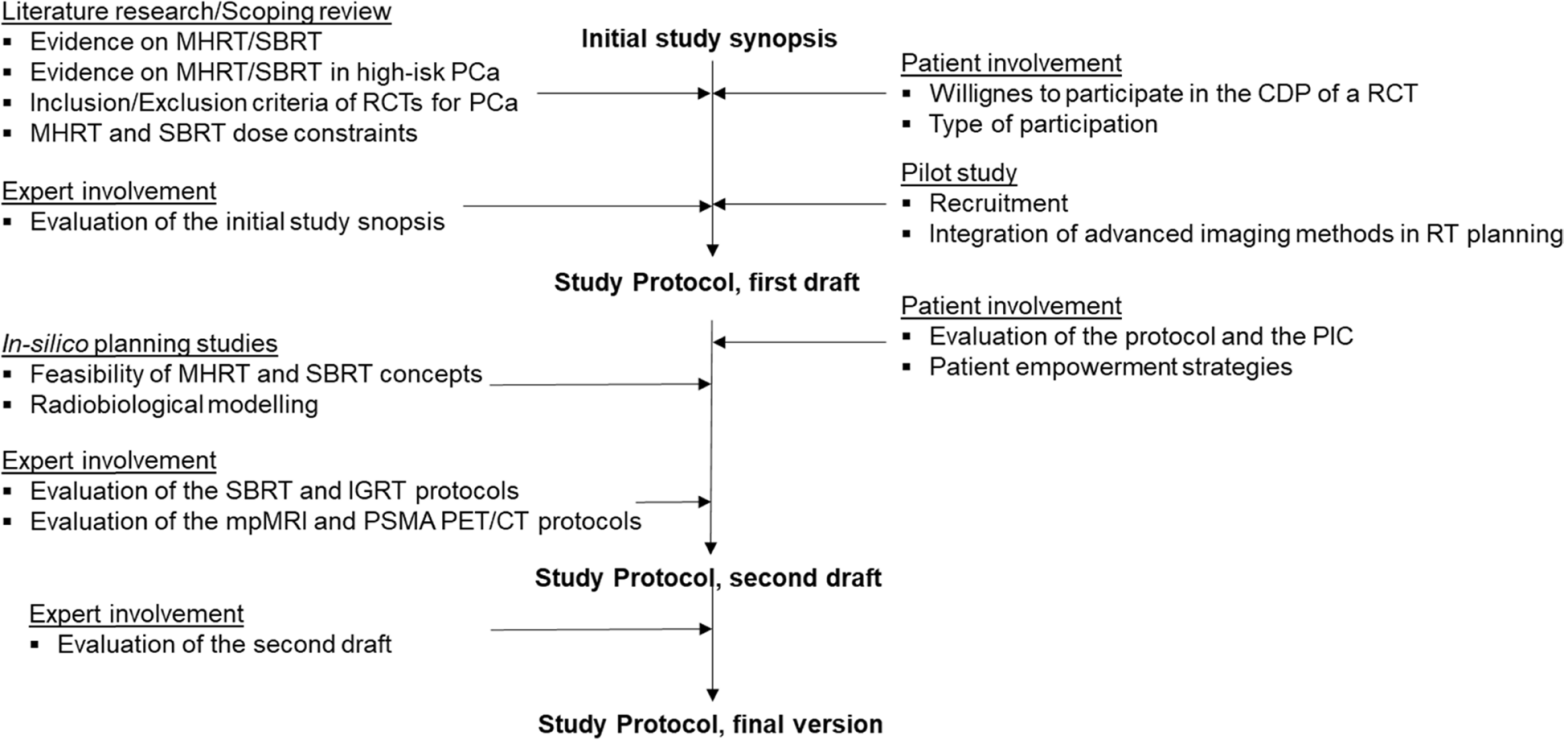
illustrates the dynamic framework of the different work packages during the concept development phase. Abbreviations: *MHRT* Moderately-hypofractionated radiotherapy, *SBRT* Stereotactic body radiotherapy, *PCa* Prostate cancer, *RCT* Randomized controlled trial, *CDP* Concept development phase, *IGRT* Image-guided radiotherapy, *PIC* Patient informed consent, *mpMRI* Multiparametric magnetic resonance imaging, *PSMA PET/CT* Prostate-specific membrane antigen positron emissions-tomography and computed tomography

During the CDP the study protocol was created and simultaneously a dynamic framework of the different WPs provided the input for modifications or clarifications (Fig. 1). Thus, upcoming results of the WPs could influence the study preparation process at several time points and at several stages of CDP. Finally, results and conclusions from all working programs ended up into a final version of the study protocol including a statistical analytic plan. This version was sent to local Ethic committees and to the *Bundesamt für Strahlenschutz* (BfS, Ministry for radiation protection, Germany) for review.

## Methodology

In the following section the applied methodology of the respective WPs will be described in detail. If not separately indicated, all methods were carried out in accordance with relevant guidelines and regulations.

### Literature searches and the scoping review

The aim of this work was to systematically identify and explore published, unpublished and ongoing studies including study protocols related to the HypoFocal-SBRT radiation treatment concepts in patients with unfavorable intermediate- or high-risk prostate cancer, through a scoping review. In contrast to a systematic review that is focusing on the question *“what works?”* and is estimating the effects for interventions, questions for a scoping review are broader focusing on *“what interventions have been studied?”*, and/or *“in what populations or settings have these interventions been studied?”*, and/or *“what outcomes have been addressed?”*. The current study pool was systematically screened to fully understand the current state of research and the context in which the treatment of patients with PCa has been studied. Therefore, this systematic approach allowed us (i) to confirm research gaps and (ii) to identify further areas appropriate for a clinical study. Furthermore, pitfalls occurring in other studies or study protocols were identified.

Systematic searches for relevant published studies were conducted on the 24th and 25th of February 2020 in Medline, Web of Science Core Collection, Cochrane Library and Science Direct (via Elsevier). Ongoing or completed unpublished studies were searched in clinicaltrials.gov and the German study register (www.drks.de). The identified lliterature was screened after predefined inclusion/exclusion criteria and key study data were extracted of studies meeting inclusion criteria. Inclusion criteria were studies with adult patients with localized prostate cancer. External beam radiotherapy (EBRT) to the prostate with focal boost was considered as intervention and EBRT to the prostate without focal boost or with different dosages were considered as comparators. Patients with distant metastases or patients under 18 years of age were excluded.

Since the final fractionation scheme for the experimental arm was determined after conduction of the scoping review, the trial team additionally performed a narrative literature research including SBRT for primary PCa. Therefore the primary databases Pubmed and EMBASE were searched using the MESH terms “prostate cancer”, “radiotherapy”, “hypofractionation”, “SBRT” and “focal dose escalation” and combining them with Boolean operators (AND, OR).

### Involvement of individual patients, patients’ representatives and patients’ self-help groups

This work was divided into two phases. In the first phase, the general patients’ willingness to participate in the preparation process and in the conduct of RCTs as well as the type of patient-participation [14] was assessed. The study team visited meetings of two local patient self-help groups and a structured teaching session (power point presentation for approximately 45 min) was conducted to explain the main aims and principles of RCTs like endpoint definition, ex−/ inclusion criteria and randomization. Only patients with biopsy-confirmed PCa were subsequently asked to fill in a questionnaire. The answers were scaled from 1 (absolutely agree) to 5 (absolutely disagree). In parallel, the study team (CZ, JW, SA and ALG) interviewed a national patient representative (EGC).

In the second phase, randomly selected PCa patients undergoing PCa radiotherapy (RT) at the Department of Radiation Oncology in Freiburg (*n* = 10), randomly selected males without cancer in the same age as potential patients (*n* = 10) and a patient representative (EGC) reviewed the study documents, focussing on the patient informed consent (PIC) forms. A questionnaire (using the same 1–5 scale as described above) was given and interviews were conducted. Finally, changes in the study documents were performed and a concept for a patient empowerment programme was designed according to the respective suggestions.

All questionnaires were anonymized and the entire work was approved by the local ethics committee of the Albert-Ludwigs-University Freiburg (No.: 20–1052). Written informed consent was obtained.

### Involvement of national and international experts and expert panels

The initial study synopsis was presented and discussed with the PCa expert panel of the German Society for Radiation Oncology (DEGRO) in January 2020. Subsequently, all members were asked to complete a questionnaire focusing on the inclusion−/exclusion criteria and the RT concept. During the CDP, the SBRT concept, the image-guidance (IGRT) concept, the multiparametric magnet resonance tomography (mpMRI) acquisition protocol and the positron emission tomography targeting prostate specific membrane antigen (PSMA PET/CT) acquisition protocol were discussed thoroughly with international and national PCa experts. Finally, an advanced version of the study synopsis was presented and discussed with the FLAME trial consortium [7, 15].

### Pilot study

The HypoFocal phase II study (DRKS00017570) started in June 2019 with two study centers (Freiburg and Berlin) and prospectively enrolled 50 patients in two study arms until January 2021. Aim of this study was to investigate the safety of PSMA-PET implementation in focal therapy planning. Therefore patients with unfavorable-intermediate and high-risk PCa and cN0/cM0 stage in mpMRI and PSMA-PET/CT were included. Patients in arm A were treated with MHRT of 60 Gy in 20 fractions and simultaneous boost to the MRI- and PET intraprostatic tumour mass (ITM) up to 75 Gy. Patients in Arm B were treated with focal dose escalated high-dose rate brachytherapy of 15 Gy to the prostate and 19 Gy to the MRI- and PET-defined ITM, followed by external beam RT to the entire prostate. The study was approved from the local ethics committee of the Albert-Ludwigs-University Freiburg (No.: 266/18) and the federal office for radiation protection in Germany (22,464/2019–003-G). Written informed consent was obtained from every patient. Endpoints of this study were analysis of safety, quality of life and evaluation of the feasibility of patient recruitment and integration of advanced imaging methods such as mpMRI and PSMA-PET/CT into the RT planning process. Potential pitfalls and problems, which occurred for the participating centers during the pilot study phase, were discussed in study meetings.

### *In-silico* radiotherapy planning studies

**Fig. 2:**
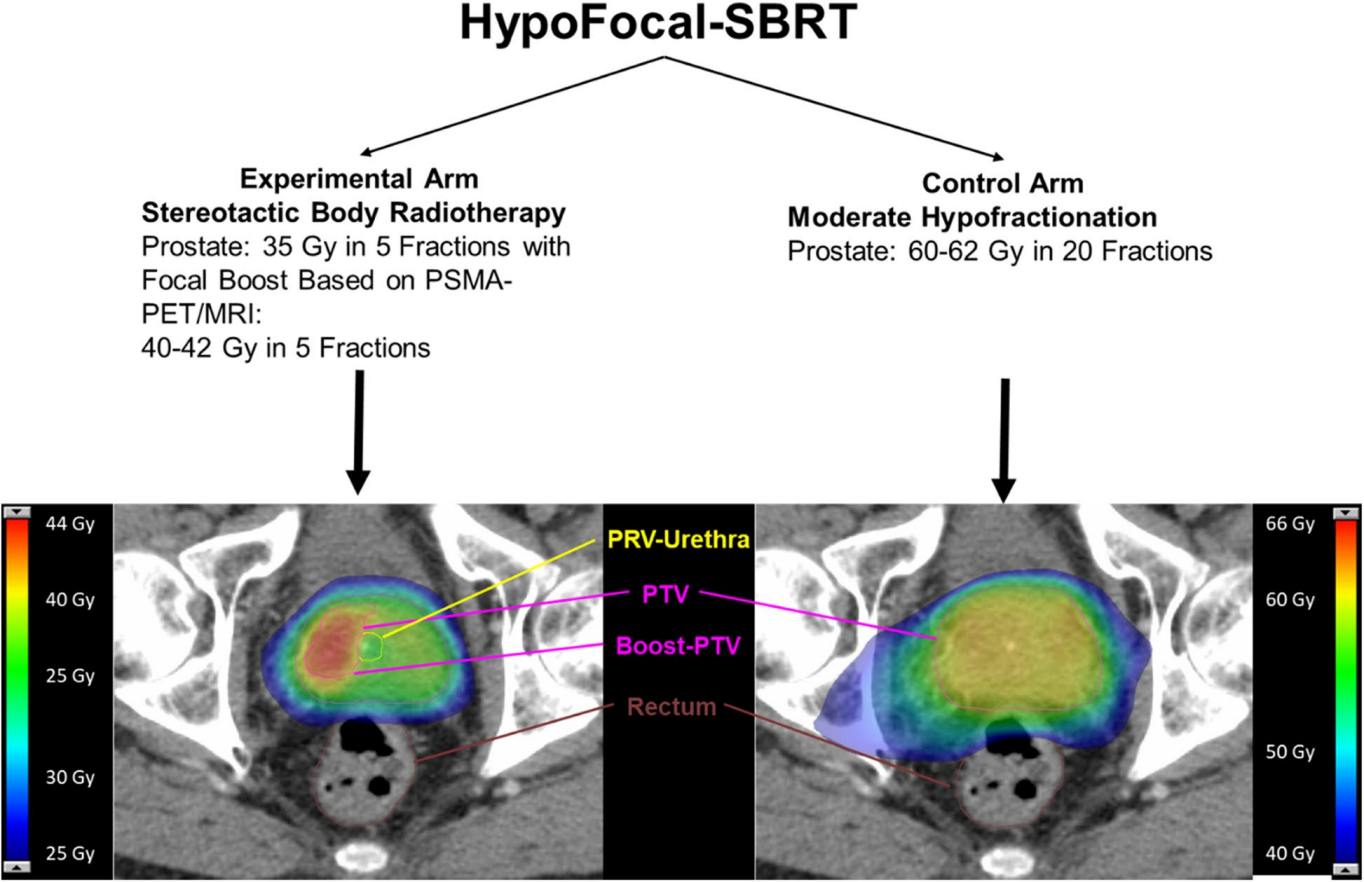
Scheme of the HypoFocal-SBRT trial and examples of focal dose escalated SBRT and MHRT. The scheme shows the design of the experimental and control arm. Details of treatment plans for focal dose escalated stereotactic body radiotherapy (SBRT) (left) and moderate hypofractionation (MHRT) (right) are shown. SBRT was planned with a prescription dose of 42 Gy to the boost planning target volume (PTV) and 35 Gy to the PTV covering the prostate in 5 fractions. MHRT was planned with a prescription dose of 62 Gy to the PTV covering the prostate. Planning organ at risk (PRV) volume of urethra was considered for boost-PTV definition. This exemplary axial slide demonstrates the steep dose gradient in focal dose escalated SBRT and the conformal homogenous MHRT. Isodoses are illustrated according to the legend

In the first step several RT dose constraints for the adjacent organs at risk (OARs) (e.g. bladder and rectum) as well as prescription doses for the target volumes (e.g. prostatic gland, intraprostatic tumor) were collected based on the results of the scoping review (2.1), a narrative literature research and the discussion with experts and expert panels (2.3). RT planning for the obtained dose constraints and prescription doses for both study arms was tested for feasibility in 15 selected cases in Eclipse v15.1 planning software (Varian, USA). Therefore, RT planning according to final dose concepts and constraints was performed in 5 cases for each dose concept (standard arm 1: MHRT with 62 Gy in 20 fractions, standard arm 2: MHRT with 60 Gy in 20 fractions and experimental arm: focal dose escalated SBRT with 35 Gy to the prostate and 42 Gy to the ITM in 5 fractions). The recently published dose constraints of the CHHiP trial for rectum were applied for MHRT [16]. Examples of SBRT and MHRT planning are included in Fig. 2.

In the second step radiobiological modelling (RM) was used to assess the therapeutic ratio of the applied RT regimen. RM enables the calculation of the tumor control probability (TCP) and the normal tissue complication probability (NTCP) *in-silico*. These models allow consequently the prediction of the potential tumor control and occurring toxicities of new RT regimen before being applied in cancer patients. The NTCP was calculated in dependence of the RT dose distribution in the organs at risk: bladder, rectum and urethra according to our previous publications [17, 18]. Additionally, the TCP was calculated under consideration of the dose distribution in the intraprostatic tumor mass in co-registered histopathology reference (19, 20). Complication free tumour control probability (P+) was calculated. Ten patients were included in this study. The study was approved from the local ethics committee of the Albert-Ludwigs-University Freiburg (No.: 469/14) and written informed consent was obtained from every patient. Please see our previous publication [18] for methodological details.

## Results

### Literature search and scoping review

In total, the literature search identified twentyone studies (nineteen completed and two ongoing studies).

Five published phase III studies focused on **MHRT** [21–25]. The target population of interest, patients at high-risk and unfavorable-intermediate risk (according to NCCNv2.2021), represented only approximately 20% of the included patients. Subgroup analysis including high risk-patients showed biochemal relapse free suvival rates (bRFS) ranging between 80.5% in the HYPRO trial [21], 83% in the trial by Arcangeli et al. [22], and 84% in th CHHIP trial [25]. MHRT was well tolerated with low rates of grade ≥ 3 toxicities and a good quality of life [21–25].

Furthermore, we identified eight published prospective studies investigating **SBRT** in primary PCa patients [5, 6, 26–31]. Again, high-risk PCa patients represented only a minority in the phase III HYPO-RT trial (approximately 11%) [6] and were not considered at all in the phase III PACE-B trial [5]. BRFS rate in the HYPO-RT-PC trial was 84% for SBRT [6]. Several phase I and II trials reported on SBRT in high-risk PCa patients, but did not reach mature follow-up time. Additionally, a series of 194 patients of which 14% were high risk, reported on insufficient 3-year bRFS rates, but used low doses with 35 G in 5 fractions [32, 33]. The phase III PACE-C trial (NCT01584258) compares conventional RT and SBRT in intermediate- and high-risk PCa patients, but recruitment is ongoing and no results have been reported yet. Thus no comprehensive picture of relapse free survival after SBRT can be obtained for this patient population. However, SBRT was well tolerated in most of the patients and may be considered as a safe treatment option associated with a good quality of life [34].

Literature searches on **focal dose escalated RT** identified two phase III studies investigating RT dose escalation in conventionally fractionated RT. Of these trials, the FLAME trial reported long term outcomes with a 7% benefit in biochemical failure free survival without significantly increasing toxicities in the experimental arm [7]. The HEIGHT trial (NCT01411332) has not yet reported any results.

Moreover, focal dose escalated MHRT was investigated in a pilot study by Onjukka et al. [35] and the phase II DELINEATE trial [36] with acceptable toxicitiy profiles. Results regarding focal dose escalated SBRT were reported by the phase II hypo-FLAME trial [37], a phase 1a/1b trial by Herrara et al. [38] and the SPARC trial [39]. The interim analysis of the Italian AIRC-IG-13218 phase II trial did not report any grade 3 or 4 toxicities after a median follow up of 17 months [40]. These studies demonstrated the feasibility of this treatment approach even with higher ablative doses and without acute ≥ grade 3 toxicities.

The scoping review and narrative literature research conducted by the trial team yielded similar results for MHRT and focal dose escalation, since neither of the two apporaches exclusively identified relevant studies (see supplemantary S1 for the scoping review) .

Additionally, prescription doses and dose constraints for MHRT and SBRT in primary PCa patients were collected and summarized by the study team under consideration of the previously depicted trials (see Table 1 for summary of dose prescriptions and constraints for SBRT). Based on this information the study team created the final dose concepts for both study arms involving an internationally renown expert (RCC, see also 3.3).

**Table 1:** summarizes dose prescriptions and constraints for stereotactic body radiotherapy as described in the current literature

Study/ Reference	Prescibed doses	Rectum					Urethra	
Herrera et al. [38]	36.25 Gy (prostate) / 45–50 Gy (boost)	V25Gy < 20 cc		D1cc < 38 Gy	D0.1cc < 41 Gy		D1cc < 39 Gy	D0.1cc < 41
Hypo-FLAME [37]	35 Gy (prostate) / 50 Gy (boost)	V28Gy < 20%	D2cc < 35 Gy	D1cc < 38 Gy	D0.035cc < 40 Gy			D0.035cc < 42 Gy
Zelefsky et al. [41]	35–40 Gy (prostate)	V24Gy < 53%*		D1cc* < 38.5 Gy	Dmax < 41.2 Gy			
PACE B [5]	36.25 Gy (prostate)	V29Gy < 20%		D1cc < 36 Gy			V42 Gy < 50%	
NRG GU005 (NCT03367702)	36.25 Gy (prostate)	D0.03cc (Gy) < = 38.06	D3cc < = 34.4	D10% < =32.63	D20% < = 29	D50% < =38.06		
	Acceptable	< 40	< 36	< 34	< 30	< 40		
American Association of Physicits in Medicine		V25Gy < 20 cc			Dmax < 38 Gy			
**Study/** **Reference**	**Bladder**				**Penile bulb**		**Small bowel**	
Herrera et al. [38]	Dmedian < 20 Gy		D1cc < 41 Gy	D0.1cc < 45				
Hypo-FLAME [37]	V28Gy < 20%	D5cc < 37 Gy	D1cc < 42 Gy		D90% < 20 Gy		D5cc < 19.5 Gy	D0.035cc < 35 Gy
Zelefsky et al. [41]	V24Gy < 53%*	D15cc < 18.3 Gy	D1cc < 42 Gy	Dmax < 42 Gy				
PACE B [5]	V18.1 Gy < 40%	D5cc < 37 Gy			V29.5 Gy < 50%			D1cc < 30 Gy
NRG GU005 (NCT03367702)	D0.03cc < =38.06	D50% < =18.12					D0.03cc < =30	
	Acceptable: < 40 Gy	< 20 Gy					< 33 Gy	
American Association of Physicits in Medicine		D15cc < 18.3 Gy		Dmax < 38 Gy	D3cc < 30 Gy	Dmax < 50 Gy	D5cc < 19.5 Gy	D0.035cc < 35 Gy

### Involvement of individual patients, patients’ representatives and patients’ self-help groups

After a teaching session on clinical trials during two meetings of local self-help groups, 30 completed questionnaires from respective PCa patients were collected. The mean age of the participants was 74 (±6.5) years and 5 (17%) patients were still working. The mean year of the initial diagnosis was 2014 (±5.5) and 12 (42%) patients received an active therapy for PCa when filling in the questionnaire. In Table 2a the patients’ willingness for participation in the preparation or conduction of a RCTs is presented. Most of the patients fully agreed that patients should participate in the CDP (*n* = 20, 66.7%) or conduction of a clinical trial (*n* = 17, 56.7%). Additionally, most of the patients fully agreed that they would participate without financial compensation (*n* = 16, 53.3%) and that a time expenditure of 2 h per month is feasible (*n* = 21, 70%). Regarding the type of participation (Table 2b), most of the patients fully agreed that they would like to have regular study progress meetings with the investigators (*n* = 15, 50%), to be involved in the definition of the study endpoints (*n* = 12, 40%) and exclusion/inclusion criteria (*n* = 8, 26.7%). In contrary, most of the patients were neutral (*n* = 12, 40%) whether patients should participate in the definition of study specific examinations. In parallel, the study team discussed the issue of patients’ participation in clinical trials with a national patient representative (EGC). This discussion also revealed a strong interest in participation in a CDP and conducting the RCT.

**Table 2:** shows the answers of patients regarding general willingness to participate in the preparation process and in the conduction of RCTs (2a) as well as the type of participation (2b)

	n total	Fully agree (1)	partially agree (2)	neutral (3)	partially disagree (4)	fully disagree (5)	No answer
**a – general willingness to participate**							
Patients should participate in the CDP of a clinical trial	30	**67%**	17%	17%	0%	0%	0%
Patients should participate in the conduction of a clinical trial	30	**57%**	27%	7%	10%	0%	0%
The involvement of patients improves the quality of clinical trials	30	**50%**	33%	13%	3%	0%	0%
I would participate in the CDP/conduction of a clinical trial without financial compensation	30	**53%**	20%	20%	3%	0%	3%
A time expenditure of 10 h per months would be feasible	30	23%	7%	7%	10%	**50%**	3%
time expenditure of 2 h per months would be feasible	30	**70%**	13%	10%	0%	7%	0%
**b – type of participation**							
Patients should be involved in definition of study endpoints	30	**40%**	17%	27%	7%	3%	7%
Patients should be involved in definition of inclusion/exclusion criteria	30	**27%**	17%	13%	23%	13%	7%
Patients should be involved in definition of study specific procedures/examinations	30	17%	30%	**40%**	3%	3%	7%
Patients should visit meet regularly with the investigators to obtain updates	30	**50%**	37%	7%	3%	0%	3%

Evaluation of the PIC by PCa patients undergoing RT demonstrated that most patients fully agreed that the actual version of the PIC is comprehensive (*n* = 6, 60%), it answers all relevant questions (*n* = 8, 80%) and is clearly structured (*n* = 7, 70%). Furthermore, most of the patients fully agreed with the asked questions (see Table 3 for details). Answers of randomly selected males not undergoing RT were similar, but the rate of full agreement was slightly lower regarding that the PIC answers all relevant questions (*n* = 5, 50%), explains personals risks of participating in the trial (*n* = 4, 40%) and explains treatment alternatives if the patient chooses not to participate in the trial (*n* = 4, 40%). Two patients (20%) and one non-irradiated male (10%) disagreed that the PIC explains treatment alternatives. See Table 3 for details. Furthermore, both groups mentioned wordings difficult to understand for laypersons, in particular the term “focal therapy”.

**Table 3:** shows the answers of the evaluation of the patient informed consent by patients undergoing radiotherapy and randomly selected males not undergoing radiotherapy (non-patients)

	In total	Fully agree (1)		Partially Agree (2)		Neutral (3)		Partially disagree (4)		Fully disagree		No answer	
The PIC …		Patients	Non-Patients	Patients	Non-Patients	Patients	Non-Patients	Patients	Non-Patients	Patients	Non-Patients	Patients	Non-Patients
is comprehensible	10	60%	50%	40%	40%	0%	10%	0%	0%	0%	0%	0%	0%
answers all relevant questions	10	80%	50%	10%	50%	10%	0%	0%	0%	0%	0%	0%	0%
is clearly structuered	10	70%	80%	30%	10%	0%	10%	0%	0%	0%	0%	0%	0%
explains the medical/therapeutic benefit of the trial	10	70%	80%	30%	W20%	0%	0%	0%	0%	0%	0%	0%	0%
explains the trial’s procedure	10	90%	70%	10%	20%	0%	10%	0%	0%	0%	0%	0%	0%
explains personal benefits of participating in the trial	10	80%	60%	20%	40%	0%	0%	0%	0%	0%	0%	0%	0%
explains personal risks of participating in the trial	10	90%	40%	10%	60%	0%	0%	0%	0%	0%	0%	0%	0%
explains treatment alternatives if the patient chooses not to participate in the trial	10	70%	40%	10%	50%	0%	0%	10%	0%	10%	10%	0%	0%
explains the legal rights of paticipating in the trial	10	80%	70%	10%	20%	0%	10%	0%	0%	0%	0%	10%	0%
contains comprehensive figures	10	60%	70%	40%	30%	0%	0%	0%	0%	0%	0%	0%	0%
containts figures, which support the presented information	10	50%	80%	50%	20%	0%	0%	0%	0%	0%	0%	0%	0%
leaves the patient without open questions	10	70%	70%	30%	10%	0%	20%	0%	0%	0%	0%	0%	0%

### Involvement of national and international experts and expert panels

The survey regarding the initial study synopsis of the DEGRO PCa expert panel was completed by 10 professionals. See Table 4 for details. The survey showed that most experts were convinced that MHRT will be the standard therapy for unfavourable-intermediate and high-risk PCa patients (*n* = 5, 56%), that patients with Gleason Score (GS) 9 should be included in the trial (*n* = 4, 44%) and that the urethra should be delineated and spared (*n* = 4, 44%). Asking whether SBRT will be the standard therapy for these patients, the expert panel was less convinced with *n* = 4 (44%) answering neutral and *n* = 4 (44%) tending to disagree. The experts did not provide uniform answers when asking whether cT3b patients should be excluded (agreeing: *n* = 3, 33%), whether administration of ADT for 6 and 18 months for unfavourable intermediate- and high-risk patients, respectively, is adequate (neutral, *n* = 4, 44%) and whether pelvic lymph nodes should electively be irradiated in patients staged cN0 with PET and MRI but high risk for nodal disease (disagreeing, *n* = 3, 33%). Most experts disagreed that unfavourable intermediate- and high-risk patients won’t be treated with MHRT, but with brachytherapy instead (*n* = 5, 56%) and that patients with cT3a stage should be excluded in this study (*n* = 6, 67%). Furthermore, the initial dose concept and dose constraints for OARs as well as specific PSA-value thresholds as inclusion criteria were thoroughly discussed. The final SBRT dose concept for target volumes and OARs and the concepts for image-guidance during SBRT were further discussed with an internationally renowned expert for PCa SBRT.

**Table 4:** shows the answers of the survey regarding the initial study synopsis of the DEGRO Prostate Cancer expert panel. The patient cohort was estimated as cT2-cT3 stage, Gleason-Score 7b-8, PSA < 40 ng/ml and cN0 cM0 stage

	In total	Fully agree (1)	partially agree (2)	neutral (3)	partially disagree (4)	fully disagree (5)	No answer
Moderate hypofractionated radiotherapy (regardless of focal dose escalation) will be the standard of care for the envisaged patient cohort in 10 ten years	9	56%	33%	11%	0%	0%	0%
Patients in the envisaged cohort will not be treated with moderate hypofractionated radiotherapy in 10 years, since all parients will be treated with SBRT	9	0%	0%	44%	44%	11%	0%
Patients in the envisaged cohort will not be treated with moderate hypofractionated radiotherapy in 10 years, since all parients will be treated with brachytherapy (at least for dose escalation purposes)	9	11%	0%	0%	33%	56%	0%
Patients with cT3a stage should be excluded in the clinical trial	9	0%	0%	22%	11%	67%	0%
Patients with cT3b stage should be excluded in the clinical trial	9	33%	11%	22%	11%	22%	0%
Patients with gleason score 9 should be excluded in the clinical trial	8	50%	13%	0%	0%	13%	25%
Duration of 6 months androgen deprivation therapy for unfavorable intermediate risk and 18 months for high risk patients is adequate	8	13%	13%	50%	0%	13%	13%
Elective lymph nodes shold be treated in patients with cN0 stage according to PET and MRI but high risk of nodal disease	7	0%	29%	29%	0%	43%	0%
Urethra should be delineated and spared	8	50%	13%	38%	0%	0%	0%
The proposed dose concept (62 Gy to the whole prostate with focal boost up to 75 Gy in 20 fractions in the experimental arm) seems reasonable	10%	10%	20%	60%	0%	0%	10%

Discussion of the final study synopsis with the FLAME-expert consortium confirmed the previous considerations. There was no relevant dissent regarding inclusion/exclusion criteria, dose concepts and RT delivery procedures.

### Pilot study

During the pilot study a total of 122 patients were screened, of which the most common reasons for exclusion, despite patients willingness to participate, were previous transurethral resection of the prostate (TURP, *n* = 15), cN+/cM+ stage after completion of staging with PSMA-PET/CT (*n* = 12) and GS = 9 (*n* = 10). Finally, 50 patients were included the HypoFocal Phase II trial between June 2019 and January 2021. In- and exclusion criteria for the HypoFoca-SBRT study were adapted based on these experiences. To properly represent high-risk PCa patients we decided to include patients with GS = 9. Since many patients were excluded due to prior prostate surgery because of urinary retention, we decided to only exclude patients who underwent such a procedure within the last 6 months prior to randomization. These experiences were considered for recruitment calculations and resulted in a envisaged enrollment of 22 patients per year and participating centre in the final HypoFocal-SBRT trial.

Median planning target volume for the focal dose escalation was 7.8 ml (IQ 5.0–11.8 ml). Median mean dose to the boost volume in the MHRT arm was 70 Gy and median D90 to the boost volume in the brachytherapy arm was 19 Gy. All patients could be treated with respect to prescription doses and dose constraints for OARs. Toxicities accordging to CTCAE v5.0 and PRO-based QOLs were assesed before, during and after therapy. Detailed results of the phase II trial will be published seperately after completion of follow up.

The conduction of the phase II trial was hampered by the Covid-19 pandemic, resulting in a reduction of face-to-face doctor-patient contact and telemedical follow ups, complicating collection of questionnaires. In addition, it revealed several pitfalls which were considered during design of the HypoFocal-SBRT study and development of the protocol: (i) Focus on patient education, also regarding compliance in terms of understanding of therapeutic procedures (e.g. preparation of bladder and rectum); (ii) Adaptive RT planning to improve RT delivery; (iii) Application of contouring recommendations for the crucial workstep of GTV contouring according to validated approaches [5, 30, 31]; (iv) detailed explanations for contouring, target volume definition, RT planning, adaptive planning and image guidance, including alternatives in cases of common errors to minimize mistakes and to provide a comprehensive manual for participating physicians and physicists; (v) Definition of a modern, central online quality assurance platform to minimize inter-observer variabilities in crucial planning steps and guarantee uniform treatments across participating centers; (vi) consideration of inter-reader variabilities and pitfalls of clinician reported toxicities [42] for finalization of the electronic case report form; (vii) close follow up with detailed assessment to adequately record relevant events (viii); (ix) establishment of clear workflows for management of toxicities; and (x) preliminary design of a translational research program, incentivizing participation of study centers and enabling the premature planning of additional research programs.

### *In-silico* radiotherapy planning studies

MHRT planning was successful in all cases. Dose prescriptions and constraints were reached in all cases, although some cases with prescription of 62 Gy had large prostates (median 60 ml, IQR 38,7–88,7 ml). Dose constraints for bladder and rectum were reached in all cases. See Table 5 for details. SBRT planning was successful in all cases and all dose prescription and constraints were reached. Boost volumes were large (median 13,2 ml, IQR 8.3–21.1 ml). Dose constraints for urethra, bladder and rectum were reached in all cases. See Table 6 for details.

**Table 5:** shows relevant metrics for prostate and organs at risk for prescription of 60 and 62 Gy in 20 fractions. Median and interquartile ranges are shown

	Prostate volume	PTV Prostate		Bladder			Rectum				
Prescription Dose		D98%	D2%	D3%	D15%	D30%	D0.01%	D5%	D22%	D38%	D57%
**60 Gy**	40.8 (29.3–46.3)	56.2 (56.2–56.8)	61.4 (61.2–61.9)	58.3 (51.3–58.9)	38.0 (32.1–42.8)	23.2 (19.8–26.5)	59.7 (59.1–60.0)	54.3 (51.6–56.5)	34.1 (30.8–36.3)	15.1 (8.3–18.6)	8.1 (6.8–8.3)
**62 Gy**	60.0 (38.7–88.7)	58.3 (58.1–58.4)	63.8 (63.5–64.0)	58.1 (54.0–59.0)	33.1 (31.9–37.1)	20.0 (17.0–22.3)	59.9 (59.8–60.1)	50.9 (49.3–55.0)	26.0 (23.5–33.6)	15.6 (13.8–18.9)	9.1 (6.4–10.8)

**Table 6:** shows relevant metrics prostate and organs at risk for prescription doses of 35 Gy to the prostate and 42 Gy to the intraprostatic tumor mass in 5 fractions. Median and interquartile ranges are shown

	Prostate volume	Boost volume (absolut / % of prostate volume)	PTV prostate	PTV boost volume		Bladder		Rectum			Urethra	
Prescition dose (Prostate/Boost)			D98%	D50%	D98%	D0.03cc	D15%	D0.03cc	D2cc	D20%	D0.01cc	D50%
**35/42 Gy**	34.5 (29.3–42-5)	13.2 (8.3–21.1) / 32 (28–55)	33.8 (33.4–33.9)	42.4 (42–42.5)	39.3 (38.3–39.9)	36.1 (35.1–36.6)	20.2 (17.3–22.2)	37.1 (36.4–37.8)	34.8 (32.7–34.8)	18.9 (16.0–21.0)	39.8 (39.5–40.2)	35.7 (35.7–36.4)

Results of RM planning study were published elsewhere [18]. Summarized prescriptions doses and constraints were reached in all plans, even when sparing of planning organ at risk volume for urethra (PRV-Urethra) was performed. Urethra-sparing reached significantly lower NTCP-Urethra values, without significantly affecting TCP based on co-registered histopathology. Consequently, complication free tumour control probability (P+) improved by urethra sparing.

## Discussion

Conceptualization and design of a RCT and its protocol is pivotal to develop the basis for a successful study. We present the conduction and results of the CDP, which resulted in the final design and study protocol of the HypoFocal-SBRT study (see Fig. 2) and [43]. To our best knowledge, this is the first publication systematically reporting on these crucial steps including insights into currently available clinical trials.

During this approximately one-year lasting process, the design of the study was shaped and modified on multiple levels to create the final protocol.

Literature searches confirmed the high clinical relevance of the aimed study, since evidence for MHRT and SBRT of high-risk PCa patients is scarce. The research performed by the trial investigators revealed the clinical need for evidence in hypofractionated RT for high-risk PCa patients, which was confirmed by the scoping review. To the best of our knowledge, no phase III RCT investigates focal dose escalated SBRT for primary PCa patients. Since (i) MHRT is the new standard treatment for localized PCa, (ii) SBRT is emerging, (iii) promising oncologic results of focal dose escalation were reported and (iv) phase I and II trials demonstrated the feasibility and tolerability of focal dose escalated SBRT, we finally decided to apply these modern RT regimes in the HypoFocal-SBRT study. Final dose concepts were developed, considering recent literature and that boost volumes derived from PET and MRI are significantly larger [12, 13] than those from previously reported trials, which only used MRI for boost definition [7, 36, 37]. Inclusion and exclusion criteria were adapted to develop patient cohorts properly filling the lack of evidence and facilitating a successful recruitment. It should be mentioned, that the scoping review added no significant new information or aspects compared to the narrative literature research performed by the trial team, which can be explained by the well-defined scenario of the envisaged study. However, the scoping review process validated independently the theoretical (current evidence of RCTs in the PCa scenario) and technical backbone (RT dose concepts) of the study protocol. Additionally, it may provide complementary information in other study scenarios. Nevertheless, a comprehensive literature search by the study team might be sufficient for study protocol development, when financial resources for independent reviews are scarce. Summarizing, an extensive literature search is imperative and should be updated throughout the CDP to take recent developments and trends into account, avoiding an outdated trial design.

Patient empowerment and involvement was a key aspect during the CPD, acknowledging the patients contribution to clinical trials and considering multiple levels of therapeutic individualization. Therefore, we integrated patients on two levels, via contacting local patients and patient groups as well as a German national patient representative. Systematic record of patients’ opinion for RCT conduction showed a high willingness to participate in RCTs and CDP of those trials. Regular progress meetings and continuous meetings with patient and patient representatives might be effective tools to overcome patient’s uncertainties. Since the PIC is a pivotal element for patient information, systematic evaluation of the PIC by patients and randomly selected males allowed us to improve deficiencies regarding treatment alternatives, risks of participation and patient-friendly wordings. In this aspect, feedback of males not undergoing RT was of particular importance since they were more critical and their knowledge of RT is similar to those of patients at initial visit. The interview with national patient representative EGC was of particular use, since an empowerment project for training of adequate bladder preparation aroused out of this cooperation. This project aims to improve patient compliance and genitourinary toxicities. Summarizing, the intensive involvement of patients and representatives significantly improved the study teams understanding of patient preferences, the quality of the PIC, and facilitated interesting cooperation. Enhancement of this work package is likely to contribute significantly to a successful patient enrolment and should be augmented with more participants in future CDPs of radiooncological trials.

Discussion with national and international expert panels greatly influenced the CDP and controversial aspects such as duration of ADT and RT of elective lymph nodes were debated, which supports the requirement of further RCTs to provide answers for distinctive clinical questions. The final design of the HypoFocal-SBRT study, in particular the development of inclusion and exclusion criteria, dose concepts, treatment delivery and image guidance benefited from these meetings. The Covid-19 pandemic has led to a rapid implementation of video conferences, which facilitates national and international conferences. Overcoming distances, we plead for a continuous and generous use of these applications in future.

The experience of the HypoFocal phase II trial suggests that the implementation of PSMA-PET into focal dose escalated RT is safe and was considered for recruitment calculations for the HypoFocal phase III trial. The recruitment experiences let the envisaged enrolment of 22 patients per year and participating center seems reasonable. The presented pitfalls were addressed in the development of the HypoFocal-SBRT study protocol resulting in a comprehensive document. The parallel conduction of the study protocol and the phase II trial led to greater focus on practicability of the HypoFoca-SBRT protocol and broadened the horizon of translational projects. Summarizing this work package was crucial for the CDP and prepared the trial team for the continuous impairment of clinical trial conduction due to the Covid-19 pandemic. This includes expansion of telemedical consultations and remote trial care.

The in-silico RT planning studies demonstrated the feasibility of the envisaged dose prescription and constraints. Stricter dose constraints proposed by the CHiPP consortium for rectum [16] were reached at 62 Gy prescription dose, enabling high-risk PCa patients the chance for higher tumor control in the standard arm and thereby an adequate control arm. Dose prescriptions and constraints were reached in all SBRT plans with focal dose escalation, despite large boost volumes, which comprised between 27 to 66% of the prostate volume. In parallel, focal dose-escalated SBRT workflows were defined aiming for reduction of treatment time and strict assessment of intrafractional organ movement. Our RM planning study supports the implementation of urethral sparing, since it bears the potential to increase the therapeutic ratio. In summary, this work package addresses relevant scientific issues and yielded encouraging results, that adequate dose distribution and escalation can be performed under consideration of strict OAR constraints, even in patients with large ITMs. However, RT planning of the envisaged study arms might benefit from larger sample cohorts and the RM study was performed with the initial trial design of dose escalated MHRT. To draw direct conclusions for focal dose escalated SBRT an additional study should be performed.

Transferring the experiences of the HypoFocal phase II trial and our RM planning studies into the clinic, implementation of PSMA-PET into focal dose escalated RT approaches will have great impact on radiotherapy planning and delivery due to the increase target volumes, but also bears great potential due to improved local staging. This supports the need for well conducted RCTs to systematically asses this question.

The process and the evaluation of a CDP has issues. First, no structured recommendations on the design and the aims of a CDP exist. Second, this process lacks measurable endpoints and criteria to objectively evaluate its effects. Thus it remains unclear, whether the CDP and the presented practical results will be reflected in improvements of study conduction and results. Consequently, we cannot evaluate the CDPs final quality at this moment. However, a possible criteria to evaluate the short-term effect of a CDP is the response of the respective ethics committees. Regarding the HypoFocal-SBRT trial the ethic committees from different participating centers had only minimal comments to the submitted protocol. The CDPs effect on patient recruitment and compliance will be evaluated after completion of the HypoFocal-SBRT trial. Critical analysis of each working program may further improve CDPs in future.

In conclusion, we systematically present the process of a one-year lasting CDP of a multicenter RCT for individualization of RT in primary PCa patients. In our experience, a dynamic framework of different interdisciplinary working programs provided significant input for the finalization of the study protocol. Consequently, a broader implementation of such CDP may enhance the robustness of RCT protocols and may decrease the failure rate. We believe that structured guidelines are warranted to accurately define the process of such CDPs in radiation oncology trials.

## Supplementary Information

**Additional file 1.**

## Data Availability

Data included in this study will be made available by contact with the corresponding author on reasonable request.
