# Supplementary material for: Feasibility, pitfalls and results of a structured concept-development phase for a randomized controlled phase III trial on radiotherapy in primary prostate cancer patients
Source: BMC Cancer. 2022 Mar 28;22:337. doi: 10.1186/s12885-022-09434-2 (PMC8960686; doi:10.1186/s12885-022-09434-2)
Supplement: Supplementary file 1 — Additional file 1. [file 12885_2022_9434_MOESM1_ESM.docx]

**Supplementary Material S1**


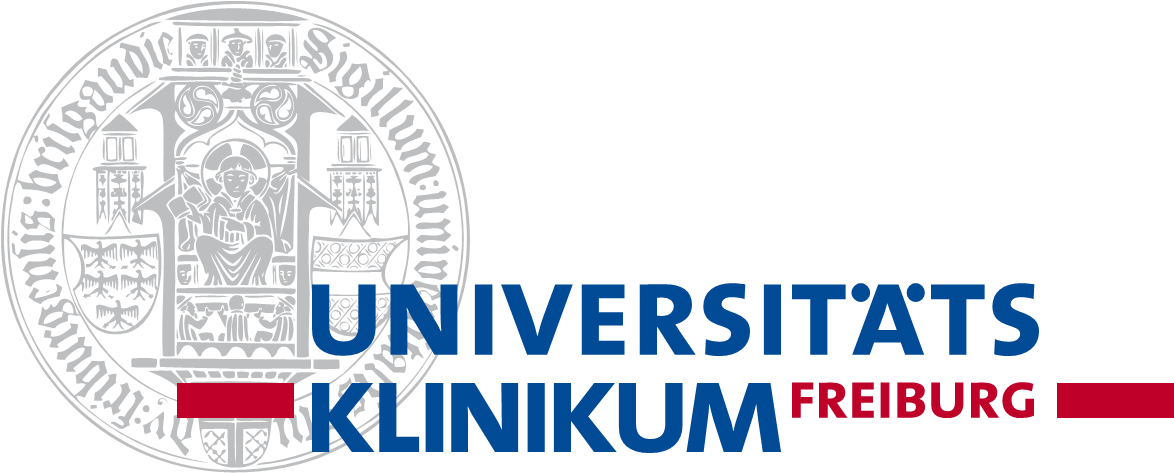


**Institute for Evidence in Medicine**

Director: Prof. Dr. med. Joerg J Meerpohl

Image-guided focal dose escalation in patients with primary prostate cancer treated with primary external beam hypofractionated radiation therapy (HypoFocal)

_________________________________

Scoping Review

Version: 1.0

Date: 14.08.2020

**Authors**

Nagavci B, Meerpohl JJ, Nothacker J, Grummich K, Schmucker C

**Affiliation**

Institute for Evidence in Medicine (for Cochrane Germany Foundation)

Medical Center - University of Freiburg

Faculty of Medicine, University of Freiburg, Germany

Breisacher Str. 86

79110 Freiburg

Content

[1 Background 4](#_Toc48141647)

[2 Aim and Objectives 4](#_Toc48141648)

[3 Methods 4](#_Toc48141649)

[3.1 Search Methods 4](#_Toc48141650)

[3.1.1 Searches for Published Studies 4](#_Toc48141651)

[3.1.2 Searches for Unpublished and Ongoing Studies 5](#_Toc48141652)

[3.1.3 Supplementary Searches 5](#_Toc48141653)

[3.1.4 Identification of Relevant Studies 5](#_Toc48141654)

[3.2 Inclusion criteria 5](#_Toc48141655)

[3.2.1 Participants/Population 5](#_Toc48141656)

[3.2.2 Intervention 5](#_Toc48141657)

[3.2.3 Comparator 6](#_Toc48141658)

[3.2.4 Relevant Outcomes 6](#_Toc48141659)

[3.2.5 Setting 6](#_Toc48141660)

[3.2.6 Study Types 6](#_Toc48141661)

[3.2.7 Extraction of Study Data 6](#_Toc48141662)

[3.2.8 Risk of Bias 7](#_Toc48141663)

[4 Results 7](#_Toc48141664)

[4.1 Results of the Literature Searches 7](#_Toc48141665)

[4.2 Characteristics of Included Studies 9](#_Toc48141666)

[5 Literature 13](#_Toc48141667)

[Appendix A 14](#_Toc48141668)

[Search Strategies 14](#_Toc48141669)

[Appendix B 17](#_Toc48141670)

[Additional studies assessed for inclusion criteria 17](#_Toc48141671)

[Appendix C 18](#_Toc48141672)

[List of included studies 18](#_Toc48141673)

# Background

This scoping review is part of the concept development phase of a prospectively planned multicenter randomized controlled trial (phase III) addressing “Image-guided focal dose escalation in patients with primary prostate cancer treated with primary external beam hypofractionated radiation therapy” (principal investigator: Prof. Dr. med. Anca-Ligia Grosu, PD Dr. med. Constantinos Zamboglou).

# Aim and Objectives

The aim of this scoping review is to systematically identify and explore published, unpublished and ongoing studies and study protocols comparing the effects of focal dose escalation to the intra tumour mass with standard prostate radiation in patients with localized prostate cancer (before conducting the proposed randomized trial). This approach will allow us to finally define and/or adapt the research question including the methodology of the randomized trial taking into account the findings e.g., research gaps and/or pitfalls in the currently available study pool addressing similar questions.

# Methods

## Search Methods

The searches for this scoping review were conducted by following the recommendation of PRESS (Peer Review of Electronic Search Strategies) [1]. Search strategies were validated by checking whether they identified studies already known. We did not use any date restrictions in the electronic searches. For each database, the date of the search, the search strategy and the number of search results were documented.

### 3.1.1 Searches for Published Studies

Systematic searches for relevant published studies were conducted on 24th and 25th of February 2020 in the following electronic data sources:

- Medline, Medline Daily Update, Medline In Process & Other Non-Indexed Citations, Medline Epub Ahead of Print (via Ovid) (the search strategy is displayed in Appendix A);
- Web of Science Core Collection: Science Citation Index-EXPANDED (SCI-EXPANDED) (via Clarivate Analytics);
- Cochrane Library (via Wiley);
- Science Direct (via Elsevier).

### 3.1.2 Searches for Unpublished and Ongoing Studies

Searches for ongoing or unpublished completed studies were performed on the 25^th^ of June 2020 in ClinicalTrials.gov ([www.clinicaltrials.gov](http://www.clinicaltrials.gov/)) and the German study register ([www.drks.de](http://www.drks.de)).

### 3.1.3 Supplementary Searches

We used relevant studies and/or systematic reviews to search for additional references via the Pubmed similar articles function (<https://www.nlm.nih.gov/bsd/disted/pubmedtutorial/020_190.html>), and forward citation tracking. Reference lists of relevant studies and systematic reviews were also reviewed.

Additional studies provided by the clinical principal investigators identified by handsearching were also assessed for inclusion (Appendix B).

### 3.1.4 Identification of Relevant Studies

Titles and abstracts of the records identified by the searches were screened by one reviewer (BN), and full texts of all potentially relevant articles were obtained. Full texts were checked for eligibility, by two reviewers and reasons for exclusions were documented (full-text screening). The complete screening process was conducted in Covidence (https://www.covidence.org/home).

## Inclusion criteria

### 3.2.1 Participants/Population

Inclusion criteria:

- Adult patients with localized prostate cancer.

Exclusion criteria:

- Prostate cancer with distant metastasis;
- Patients under 18 years of age.

### 3.2.2 Intervention

We considered the following interventions:

- External beam radiation therapy to the prostate with focal boost (to the intra prostatic tumour mass).

### Comparator

We considered the following comparator:

- External beam radiation therapy to the prostate without focal boost (to the intra prostatic tumour mass);
- External beam radiation therapy to the prostate with focal boost (to the intra prostatic tumour mass) in different dosages than the intervention.

### 3.2.4 Relevant Outcomes

Any outcomes reported in the eligible study pool.

### 3.2.5 Setting

Any setting was included.

### 3.2.6 Study Types

Inclusion criteria:

- Randomized controlled trials;
- Non-randomized controlled studies of interventions (NRSI; using strategies of non-random allocation for assigning interventions).

Exclusion criteria:

- Phase I studies;
- Studies without a control group;
- Case reports;
- Case series;
- Review articles and clinical guidelines (were excluded but reviewed for relevant studies);
- Work that has not been peer-reviewed (e.g., thesis, editorials, letters, comments);
- Observational/retrospective studies.

We did not apply any exclusion criteria regarding study duration.

### 3.2.7 Extraction of Study Data

The following study data were extracted and relevant information tabulated:

- Study characteristics, i.e., author, year of publication, study type, start and end of study, sample size, follow-up time;
- Setting, i.e., geographical and organizational setting;
- Characteristics of the participants;
- Characteristics of the intervention;
- Characteristics of the comparator;
- Reported outcomes and their definitions.

Data from each included study were extracted by one reviewer (BN) and checked by a second (CS). Disagreements were resolved through discussion until consensus was reached.

### 3.2.8 Risk of Bias

Risk of bias was not assessed [2].

# 4 Results

### 4.1 Results of the Literature Searches

The electronic searches identified 9830 records (references), including 3374 duplicates. Among the 6456 unique records screened, 481 were considered for full-text screening. Of these, four studies corresponding to seven publications were eligible for inclusion (Appendix C):

- three RCTs (one completed, two ongoing, corresponding to five publications);
- one NRSI (completed corresponding to two publications).

Table 1. Results of the database searches.

| **Database** | **Hits** |
| --- | --- |
| Medline | 3580 |
| Cochrane Library | 1072 |
| Web of Science | 2942 |
| Science Direct | 2224 |
| Trial registers | 11 |
| Supplementary searches | 1 |
| **Total** | **9830** |
| Duplicates* | 3374 |
| **Total after removing duplicates** | **6456** |

*Automatically identified by deduplication function (3083 via Endnote, 291 via Covidence)

Figure 1: Results of bibliographic literature search and study selection (PRISMA flow diagram)^ꝉ^.


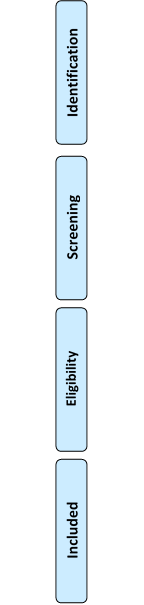


Supplementary searches

(n=1)

Records identified through database searching
(n=9830)

Records after duplicates removed

(n=6456)

Excluded at abstract level

(n=5975)

Records screened

(n=6456)

Full-text articles excluded (n=474**)**

Ineligible intervention: 449

Ineligible population: 3

Reviews: 1

Guidelines/statements: 8

Single arm studies: 1

Abstracts**: 3

Commentaries: 1

Duplicates: 8

Full-text articles assessed for eligibility

(n=481)

Records included

(n=7)*

*four studies included, with a total of seven published reports.

**Abstracts of included studies, no additional results were reported.

__________________________________________________________________________________

^ꝉ^ Moher D, Liberati A, Tetzlaff J, Altman DG, The PRISMA Group (2009). Preferred Reporting Items for Systematic Reviews and Meta-Analyses: The PRISMA Statement. PLoS Med 6(7): e1000097. doi:10.1371/journal.pmed1000097; For more information, visit www.prisma-statement.org.

### 4.2 Characteristics of Included Studies

**Table 2a.** Characteristics of published studies (n=2).

| **Author/**  **ACRONYM** | **Study design**  **(N centers)** | **Country /**  **Recruitment time** | **Definition of patient population** |  | **Radiation localization** | **Radiation technique** | **Organ dose**  **(fractions)** | **Focal boost dose (fractions)** | **Imaging modality** | **N randomized** | **Age in years**  **(range)** | **Median follow-up**  **(years)** |
| --- | --- | --- | --- | --- | --- | --- | --- | --- | --- | --- | --- | --- |
| **Monninkhof 2018 [3-5]**  **(FLAME)** | Phase III RCT  (3) | Netherlands/  Belgium  2009-15 | Prostate cancer patients with **intermediate risk** (if one of the following is present: T2 carcinoma, or the Gleason score = 7, or iPSA 10–20 ng/mL), or **high-risk** (if one or more of the following factors are present: T3 carcinoma, or the Gleason score > 7, or iPSA > 20 ng/mL.) as defined by the Ash et al. criteria [6]. Additionally defined also by NCCN criteria. | I | Prostate with focal boost | IMRT/  VMAT | 77Gy  (35 x 2.2Gy) | 95 Gy  (35 x 2.7Gy) | MRI | 284 | Mean  70  (46-83) | 4.5 |
|  |  |  |  | C | Prostate |  |  | none |  | 287 | Mean  70  (47-85) |  |
| **Murray 2020 [7, 8]**  **(DELINEATE)** | Phase II  Prospective NRSI (cohort study)  (1) | United Kingdom  2011-15 | Patients with **intermediate-risk or high-risk** local prostate adenocarcinoma, defined according to NCCN. | I | Prostate with focal boost | IMRT | 74Gy  (37 x 2Gy) | 82Gy  (37 x 2.2Gy) | MRI | 55 | Median  70  (57-80) | 6.2 |
|  |  |  |  | C |  |  | 60Gy  (20 x 3Gy) | 67Gy  (20 x 3.35Gy) |  | 50 | Median  71.5  (67-79) | 4.3 |

C: Control, I: Intervention, IMRT: Intensity-modulated Radiation Therapy, iPSA: Initial Prostate Specific Antigen, N: Number; NA: Not available, NCCN: National Comprehensive Cancer Network criteria, NRS: Non-randomized controlled study of intervention, RCT: Randomized Controlled Trial, VMAT: Volumetric Modulated Arc Therapy.

| **Author/**  **ACRONYM** | **Study design**  **(N centers)** | **Country /**  **Recruitment time** | **Definition of patient population** |  | **Radiation localization** | **Radiation technique** | **Organ dose**  **(fractions)** | **Focal boost dose (fractions)** | **Imaging modality** | **Planned N** | **Age in years (range)** | **Planned**  **Follow-up (years)** |
| --- | --- | --- | --- | --- | --- | --- | --- | --- | --- | --- | --- | --- |
| **HEIGHT Trial [9]** | Phase III  RCT  (NA) | United States  2011-NA | Patients with localized adenocarcinoma of the prostate with T1-T3a, Gleason score 6-8, PSA ≤100 ng/mL, Zubrod performance status <2 | I | Prostate with focal boost | IMRT | 76Gy  (38x2Gy) | 91.2 Gy  (38x2.4Gy) | MRI | NA | NA | 5.3 |
|  |  |  |  | C | Prostate |  | 80Gy  (40x2Gy) | none |  |  |  |  |
| **PIVOTALBOOST Trial [10]** | Phase III  RCT  (18) | United Kingdom  2018-22 | Patients with histologically confirmed, non-metastatic adenocarcinoma of the prostate, belonging to NCCN **intermediate risk** (T2b-c N0M0, and/or Gleason 3+4 and /or PSA 10-20 ng/ml), or NCCN **high risk** (T3a, T3b or T4 N0M0 and/or dominant Gleason 4 or 5 and/or PSA >20) | Ia | Prostate pelvic and focal boost | IMRT | NA | NA | MRI | 1952 | NA | 10 |
|  |  |  |  | Ib | Prostate and pelvic |  |  |  |  |  |  |  |
|  |  |  |  | Ic | Prostate with focal boost | IMRT  (focal boost with IMRT or brachy-therapy) |  |  |  |  |  |  |
|  |  |  |  | C | Prostate |  |  |  |  |  |  |  |

**Table 2b.** Characteristics of ongoing studies (protocols, n=2).

C: Control, I: Intervention, IMRT: Intensity-modulated Radiation Therapy, iPSA: Initial Prostate Specific Antigen, N: Number; NA: Not available, NCCN: National Comprehensive Cancer Network criteria. RCT: Randomized Controlled Trial.

**Table 3.** Outcomes considered in the identified studies.

| **Author/**  **ACRONYM** | **Reported outcomes** | **Time-point (when assessed)** | **Definition/Measure** |
| --- | --- | --- | --- |
| **Monninkhof 2018**  **[3-5]**  **(FLAME)** | Biochemical-progression-free survival  (primary outcome) | 5 years - but follow-up time insufficient to report this outcome. | PSA concentration greater than the nadir plus 2 ng/mL (Phoenix definition). |
|  | Treatment related toxicity (acute and late) | Acute toxicity assessed weekly during treatment and 4 weeks after treatment.  Late toxicity was planned to be assessed every 6 months until 10 years after treatment (as per protocol).  Toxicity scores were presented up to 2 years after the first radiation treatment. | Collected using CTC for adverse events version 3.0, including urinary frequency/urgency, urinary retention, bladder spasms, urinary incontinence, GU hemorrhage, dysuria, rectal or perirectal pain, proctitis, diarrhea, flatulence, hemorrhoids, anal incontinence, rectal fistula and rectal hemorrhage. |
| **Murray 2020**  **[7, 8]**  **(DELINEATE)** | Late RTOG rectal toxicity  (primary outcome) | 1 year | Cumulative late rectal toxicity of grade 2 or worse, calculated using Kaplan-Meier methods. |
|  | Treatment related toxicity (acute and late), measured with clinician- and patient-reported outcomes | Clinician-reported outcomes were assessed pre-hormone and pre-radiation therapy.  Clinical assessment of acute toxicity was made weekly until week 8; then at weeks 10, 12, and 18 (from the start of radiation therapy).  Clinical assessment of late toxicity was assessed at 6 months from the start of radiation therapy and then every 6 months up to 5 years.  Patient reported outcomes assessed at enrollment, before radiation therapy, at week 18, and then at 6, 12, 18, 24 months from start of radiation therapy. | Clinician-reported outcomes assessed using National Cancer Institute CTC for Adverse Events v4 grading [11], Royal Marsden Hospital grading [12], and Gulliford rectal scores [13]).  Clinical assessment of acute toxicity using RTOG scoring system [14]).  Clinical assessment of late toxicity using RTOG, NCI CTCAE v4, Gulliford rectal, and RMH scoring systems.  Patient-reported outcomes assessed using modified inflammatory bowel disease[15], Vaizey[16], International Prostate Symptom Score [17], Expanded Prostate Cancer Index Composite-26 questionnaires. |
|  | Time to biochemical progression | PSA was evaluated at weeks 10 and 18 from the start of radiation therapy, at 6 months, and then every 6 months for 5 years. | Defined as an increase in serum PSA of at least 2ng/mL greater than the post-radiation therapy nadir and confirmed with a second consecutive reading of at least 2 ng/mL greater than the post-treatment nadir. |
| **HEIGHT Trial**  **Ongoing**  **[9]** | Rate of prostate biopsy positivity  (primary outcome) | 2 years (post treatment) | |
|  | Acute and late toxicity | Acute toxicity: toxicity occurring during treatment and ≤ 3 months of completing treatment.  Late toxicity: toxicity occurring > 3 months after treatment completion. | |
|  | Quality of life | Up to 5.3 years (unspecified schedule) | Health-related quality of life, prostate cancer-specific anxiety and prostate cancer-specific quality of life as measured by psychosocial questionnaires. |
|  | Incidence and relationship of circulating free DNA and tumor cells to tissue biomarkers and prostate biopsy positivity | 2 years (post treatment) | |
|  | Quantification of biomarker expression in different prostate tumor regions | Up to 5.3 years (unspecified schedule) | In different prostate tumor regions, comparing specifically the functional MRI suspicious regions to those that are not suspicious. |
|  | Biochemical failure |  | PSA ≥ nadir + 2 ng/mL |
|  | Clinical failure |  | At least local failure due to newly identified extension outside of the prostate after initial regression, or urinary obstructive symptoms with carcinoma found at TURP or regional/distant failure due to radiographic evidence metastasis (nodal or hematogenous spread). |
|  | Failure-Free Survival |  | Elapsed time from start of radiotherapy to first documented evidence of biochemical or clinical failure or death from any cause, whichever occurs first; in the absence of any event defining failure, follow-up time will be censored at the date of last documented failure-free status. |
|  | Rate of Overall Survival |  | The elapsed time from start of radiotherapy to death from any cause. For surviving patients, follow-up will be censored at the date of last contact. |
| **PIVOTALBOOST Trial**  **Ongoing**  **[10]** | Failure-free survival  (primary endpoint) | Up to 10 years | Measured by the time to first biochemical failure, recommencement of androgen deprivation therapy, local recurrence, lymph node/pelvic recurrence, distant metastases or death due to prostate cancer. |
|  | Time to loco-regional recurrence |  | Time to biochemical failure or prostate recurrence; metastatic relapse free survival; overall and prostate cancer specific survival; time to recommencement of androgen deprivation therapy is measured. |
|  | Acute bladder and bowel toxicity | At 3 months | Measured using RTOG and CTC (v4.0) |
|  | Late toxicity | Up to 10 years | Measured using RTOG and CTC (v4.0) |
|  | Health economic endpoints |  | Measured using EQ-5D |
|  | Quality of life |  | Measured using ALERT-B screening tool, Gastrointestinal Symptom Rating Scale, IIEF-5 Questionnaire, International Prostate Symptom Score, and Expanded Prostate Index Composite-26 Short Form questionnaire. |

ALERT-B: Assessment of Late Effects of RadioTherapy – Bowel; CTC: Common Terminology Criteria for Adverse Events, PSA: Prostate Specific Antigen; RTOG: Radiation Therapy Oncology Group; TURP: transurethral resection of the prostate.

# 5 Literature

1. McGowan, J.S., M.; Salzwedel, D.M.; Cogo, E.; Foerster, V.; Lefebvre, C., *PRESS Peer Review of Electronic Search Strategies: 2015 Guideline Statement.* Journal of Clinical Epidemiology., 2016. **56**(10): p. 1390-1397.

2. Schmucker, C., et al., *[Methods of evidence mapping. A systematic review].* Bundesgesundheitsblatt Gesundheitsforschung Gesundheitsschutz, 2013. **56**(10): p. 1390-7.

3. Lips, I.M., et al., *Single blind randomized phase III trial to investigate the benefit of a focal lesion ablative microboost in prostate cancer (FLAME-trial): study protocol for a randomized controlled trial.* Trials [Electronic Resource], 2011. **12**: p. 255.

4. Monninkhof, E.M., et al., *Standard whole prostate gland radiotherapy with and without lesion boost in prostate cancer: toxicity in the FLAME randomized controlled trial.* Radiotherapy and Oncology, 2018. **127**(1): p. 74‐80.

5. Nct. *FLAME: Investigate the Benefit of a Focal Lesion Ablative Microboost in Prostate Cancer (FLAME).* . 2010; Available from: <https://clinicaltrials.gov/ct2/show/NCT01168479>

6. Ash, D., et al., *ESTRO/EAU/EORTC recommendations on permanent seed implantation for localized prostate cancer.* Radiother Oncol, 2000. **57**(3): p. 315-21.

7. Murray, J.R., et al., *Standard and Hypofractionated Dose Escalation to Intraprostatic Tumor Nodules in Localized Prostate Cancer: Efficacy and Toxicity in the DELINEATE Trial.* International Journal of Radiation Oncology*Biology*Physics, 2020. **106**(4): p. 715-724.

8. Isrctn. *Dose escalation to intraprostatic tumour nodules in localised prostate cancer.* 2011; Available from: <https://doi.org/10.1186/ISRCTN04483921>.

9. Nct. *Hypofractionated Image-Guided Radiotherapy For Prostate Cancer: the HEIGHT Trial.* 2011; Available from: <https://clinicaltrials.gov/ct2/show/NCT01411332>.

10. Shama Hassan. *A phase III randomised controlled trial of prostate and pelvis versus prostate alone radiotherapy with or without prostate boost.* 2018; Available from: <https://www.isrctn.com/ISRCTN80146950>.

11. Trotti, A., et al., *CTCAE v3.0: development of a comprehensive grading system for the adverse effects of cancer treatment.* Semin Radiat Oncol, 2003. **13**(3): p. 176-81.

12. Dearnaley, D.P., et al., *The early toxicity of escalated versus standard dose conformal radiotherapy with neo-adjuvant androgen suppression for patients with localised prostate cancer: results from the MRC RT01 trial (ISRCTN47772397).* Radiother Oncol, 2007. **83**(1): p. 31-41.

13. Gulliford, S.L., et al., *A comparison of dose-volume constraints derived using peak and longitudinal definitions of late rectal toxicity.* Radiotherapy and oncology : journal of the European Society for Therapeutic Radiology and Oncology, 2010. **94**(2): p. 241-247.

14. Pilepich, M.V., et al., *Correlation of radiotherapeutic parameters and treatment related morbidity--analysis of RTOG Study 77-06.* Int J Radiat Oncol Biol Phys, 1987. **13**(7): p. 1007-12.

15. Guyatt, G., et al., *A new measure of health status for clinical trials in inflammatory bowel disease.* Gastroenterology, 1989. **96**(3): p. 804-10.

16. Vaizey, C.J., et al., *Prospective comparison of faecal incontinence grading systems.* Gut, 1999. **44**(1): p. 77-80.

17. Barry, M.J., et al., *The American Urological Association symptom index for benign prostatic hyperplasia. The Measurement Committee of the American Urological Association.* J Urol, 1992. **148**(5): p. 1549-57; discussion 1564.

# Appendix A

### Search Strategies

**Medline (Ovid)**

Ovid MEDLINE(R) and Epub Ahead of Print, In-Process & Other Non-Indexed Citations and Daily 1946 to February 24, 2020

| # | Searches | Results |
| --- | --- | --- |
| 1 | exp Prostatic Neoplasms/ | 125447 |
| 2 | (Prostat* adj3 cancer).ti,ab,kf. | 121865 |
| 3 | (prostat* adj3 adenocarcinoma).ti,ab,kf. | 7717 |
| 4 | (prostat* adj3 (tumour or tumor)).ti,ab,kf. | 7503 |
| 5 | (prostat* adj3 neoplas*).ti,ab,kf. | 7628 |
| 6 | 1 or 2 or 3 or 4 or 5 | 158690 |
| 7 | exp Radiotherapy, Image-Guided/ | 2951 |
| 8 | exp Radiosurgery/ | 15168 |
| 9 | exp Dose Fractionation, Radiation/ | 9589 |
| 10 | Hypofraction*.ti,ab,kf. | 3426 |
| 11 | (fraction* adj3 radi*).ti,ab,kf. | 10844 |
| 12 | (dos* adj3 fraction*).ti,ab,kf. | 9941 |
| 13 | (dos* adj3 escalat*).ti,ab,kf. | 18457 |
| 14 | (Boost* adj3 radi*).ti,ab,kf. | 1359 |
| 15 | Stereotactic.ti,ab,kf. | 24803 |
| 16 | 7 or 8 or 9 or 10 or 11 or 12 or 13 or 14 or 15 | 73084 |
| 17 | 6 and 16 | 4170 |
| 18 | exp animals/ not exp humans/ | 4671550 |
| 19 | 17 not 18 | 4136 |
| 20 | editorial/ | 518586 |
| 21 | letter/ | 1063632 |
| 22 | Congress/ | 65972 |
| 23 | 20 or 21 or 22 | 1645911 |
| 24 | 19 not 23 | 3971 |
| 25 | limit 24 to yr="2000 -Current" | 3731 |
| 26 | limit 25 to (english or german) | 3580 |

**Cochrane Library (via Wiley)**

| ID | Search | Hits |
| --- | --- | --- |
| #1 | MeSH descriptor: [Prostatic Neoplasms] explode all trees | 5396 |
| #2 | prostat* NEAR/3 cancer | 13763 |
| #3 | prostat* NEAR/3 adenocarcinom* | 803 |
| #4 | prostat* NEAR/3 tumor | 691 |
| #5 | #1 or #2 or #3 or #4 | 14470 |
| #6 | MeSH descriptor: [Radiotherapy Dosage] explode all trees | 2490 |
| #7 | MeSH descriptor: [Radiosurgery] explode all trees | 212 |
| #8 | hypofraction* | 940 |
| #9 | dos* NEAR/3 fraction* | 2301 |
| #10 | fraction* NEAR/3 radi* | 2503 |
| #11 | dos* NEAR/3 escalat* | 11776 |
| #12 | boost* NEAR/3 radi* | 351 |
| #13 | stereotactic | 2014 |
| #14 | #6 or #7 or #8 or #9 or #10 or #11 or #12 or #13 | 18760 |
| #15 | #5 and #14 | 1214 |
| #16 | MeSH descriptor: [Animals] explode all trees | 15784 |
| #17 | MeSH descriptor: [Humans] explode all trees | 8478 |
| #18 | #16 not #17 |  |
| #19 | #15 not #18 with Publication Year from 2000 to 2020, in Trials | 1072 |

**Web of Science Core Collection: Science Citation Index-EXPANDED (SCI-EXPANDED) (via Clarivate Analytics)**

| # 11 | [2,942](https://apps.webofknowledge.com/summary.do?product=WOS&doc=1&qid=103&SID=F35awPzMLLjw4QPySj5&search_mode=AdvancedSearch&update_back2search_link_param=yes) | (#7 OR #6) *AND***LANGUAGE:** (English OR German)  **Refined by:** **WEB OF SCIENCE CATEGORIES:** ( ONCOLOGY OR RADIOLOGY NUCLEAR MEDICINE MEDICAL IMAGING OR UROLOGY NEPHROLOGY OR MEDICINE GENERAL INTERNAL ) AND **DOCUMENT TYPES:** ( ARTICLE OR EARLY ACCESS OR REVIEW )  *Indexes=SCI-EXPANDED Timespan=2000-2020* |
| --- | --- | --- |
| # 10 | [3,967](https://apps.webofknowledge.com/summary.do?product=WOS&doc=1&qid=102&SID=F35awPzMLLjw4QPySj5&search_mode=AdvancedSearch&update_back2search_link_param=yes) | (#7 OR #6) *AND***LANGUAGE:** (English OR German)  **Refined by:** **WEB OF SCIENCE CATEGORIES:** ( ONCOLOGY OR RADIOLOGY NUCLEAR MEDICINE MEDICAL IMAGING OR UROLOGY NEPHROLOGY OR MEDICINE GENERAL INTERNAL )  *Indexes=SCI-EXPANDED Timespan=2000-2020* |
| # 9 | [4,254](https://apps.webofknowledge.com/summary.do?product=WOS&doc=1&qid=101&SID=F35awPzMLLjw4QPySj5&search_mode=AdvancedSearch&update_back2search_link_param=yes) | (#7 OR #6) *AND***LANGUAGE:** (English OR German)  *Indexes=SCI-EXPANDED Timespan=2000-2020* |
| # 8 | [4,540](https://apps.webofknowledge.com/summary.do?product=WOS&doc=1&qid=97&SID=F35awPzMLLjw4QPySj5&search_mode=AdvancedSearch&update_back2search_link_param=yes) | #7 OR #6  *Indexes=SCI-EXPANDED Timespan=All years* |
| # 7 | [4,490](https://apps.webofknowledge.com/summary.do?product=WOS&doc=1&qid=96&SID=F35awPzMLLjw4QPySj5&search_mode=AdvancedSearch&update_back2search_link_param=yes) | #5 NOT #3  *Indexes=SCI-EXPANDED Timespan=All years* |
| # 6 | [58](https://apps.webofknowledge.com/summary.do?product=WOS&doc=1&qid=95&SID=F35awPzMLLjw4QPySj5&search_mode=AdvancedSearch&update_back2search_link_param=yes) | #4 AND #1  *Indexes=SCI-EXPANDED Timespan=All years* |
| # 5 | [4,897](https://apps.webofknowledge.com/summary.do?product=WOS&doc=1&qid=94&SID=F35awPzMLLjw4QPySj5&search_mode=AdvancedSearch&update_back2search_link_param=yes) | #2 AND #1  *Indexes=SCI-EXPANDED Timespan=All years* |
| # 4 | [87](https://apps.webofknowledge.com/summary.do?product=WOS&doc=1&qid=93&SID=F35awPzMLLjw4QPySj5&search_mode=AdvancedSearch&update_back2search_link_param=yes) | TS=(Brachytherapy NEAR/5 Hypofraction*)  *Indexes=SCI-EXPANDED Timespan=All years* |
| # 3 | [16,546](https://apps.webofknowledge.com/summary.do?product=WOS&doc=1&qid=92&SID=F35awPzMLLjw4QPySj5&search_mode=AdvancedSearch&update_back2search_link_param=yes) | TI=(Brachytherapy)  *Indexes=SCI-EXPANDED Timespan=All years* |
| # 2 | [76,048](https://apps.webofknowledge.com/summary.do?product=WOS&doc=1&qid=91&SID=F35awPzMLLjw4QPySj5&search_mode=AdvancedSearch&update_back2search_link_param=yes) | TS=("Dos* Fractionation" OR (hypofraction* NEAR/3 (radiotherapy OR dosage OR therapy)) OR (dos* NEAR/1 fraction*) OR (fraction* NEAR/3 radi*) OR (dos* NEAR/3 escalat*) OR stereotactic)  *Indexes=SCI-EXPANDED Timespan=All years* |

**Science Direct (via Elsevier)**

| Title, abstract, keywords: (prostate) AND (fractionation OR fractionated OR hypofractionated OR hypofractination OR escalation OR boost) |
| --- |
| Limits:  Year(s) 2000-2020  Article types: Review articles, Research articles, Practice guidelines |
| 2,224 results |

# Appendix B

### Additional studies assessed for inclusion criteria

These studies were identified by the study investigators and were assessed for inclusion criteria:

| Incrocci L, Wortel RC, Alemayehu WG, Aluwini S, Schimmel E, Krol S, et al. Hypofractionated versus conventionally fractionated radiotherapy for patients with localised prostate cancer (HYPRO): final efficacy results from a randomised, multicentre, open-label, phase 3 trial. The Lancet Oncology. 2016;17(8):1061-9. |
| --- |
| Lee WR, Dignam JJ, Amin MB, Bruner DW, Low D, Swanson GP, et al. Randomized Phase III Noninferiority Study Comparing Two Radiotherapy Fractionation Schedules in Patients With Low-Risk Prostate Cancer. Journal of Clinical Oncology. 2016;34(20):2325-32. |
| Dearnaley D, Syndikus I, Mossop H, Khoo V, Birtle A, Bloomfield D, et al. Conventional versus hypofractionated high-dose intensity-modulated radiotherapy for prostate cancer: 5-year outcomes of the randomised, non-inferiority, phase 3 CHHiP trial. The Lancet Oncology. 2016;17(8):1047-60. |
| Catton CN, Lukka H, Gu C-S, Martin JM, Supiot S, Chung PWM, et al. Randomized Trial of a Hypofractionated Radiation Regimen for the Treatment of Localized Prostate Cancer. Journal of Clinical Oncology. 2017;35(17):1884-90. |
| Arcangeli G, Saracino B, Arcangeli S, Gomellini S, Petrongari MG, Sanguineti G, et al. Moderate Hypofractionation in High-Risk, Organ-Confined Prostate Cancer: Final Results of a Phase III Randomized Trial. Journal of Clinical Oncology. 2017;35(17):1891-7. |

# Appendix C

### List of included studies

| **Included studies (n=4)** | **Corresponding publications / protocols (n=7)** |
| --- | --- |
| Monninkhof 2018 | Lips IM, van der Heide UA, Haustermans K, van Lin EN, Pos F, Franken SP, et al. Single blind randomized phase III trial to investigate the benefit of a focal lesion ablative microboost in prostate cancer (FLAME-trial): study protocol for a randomized controlled trial. Trials [Electronic Resource]. 2011;12:255. |
|  | Monninkhof EM, van Loon JWL, van Vulpen M, Kerkmeijer LGW, Pos FJ, Haustermans K, et al. Standard whole prostate gland radiotherapy with and without lesion boost in prostate cancer: toxicity in the FLAME randomized controlled trial. Radiotherapy and Oncology. 2018;127(1):74‐80. |
|  | Nct. FLAME: Investigate the Benefit of a Focal Lesion Ablative Microboost in Prostate Cancer (FLAME). 2010. Available from: <https://clinicaltrials.gov/ct2/show/NCT01168479> |
| Murray 2020 | Murray JR, Tree AC, Alexander EJ, Sohaib A, Hazell S, Thomas K, et al. Standard and Hypofractionated Dose Escalation to Intraprostatic Tumor Nodules in Localized Prostate Cancer: Efficacy and Toxicity in the DELINEATE Trial. International Journal of Radiation Oncology*Biology*Physics. 2020;106(4):715-24. |
|  | Isrctn. Dose escalation to intraprostatic tumour nodules in localised prostate cancer 2011. Available from: https://doi.org/10.1186/ISRCTN04483921. |
| HEIGHT Trial | Ongoing  Nct. Hypofractionated Image-Guided Radiotherapy For Prostate Cancer: the HEIGHT Trial 2011. Available from: https://clinicaltrials.gov/ct2/show/NCT01411332. |
| PIVOTALBOOST Trial | Ongoing  Shama Hassan. A phase III randomised controlled trial of prostate and pelvis versus prostate alone radiotherapy with or without prostate boost: ISRCTN; 2018. Available from: https://www.isrctn.com/ISRCTN80146950. |
